# Denoising and Simplification of 3D Scan Data of Damaged Aero-Engine Blades for Accurate and Efficient Rigid and Non-Rigid Registration

**DOI:** 10.3390/s25196148

**Published:** 2025-10-04

**Authors:** Hamid Ghorbani, Farbod Khameneifar

**Affiliations:** 1 Department of Mechanical Engineering, McGill University, Montreal, QC H3A 0G4, Canada; 2 Aerospace Manufacturing Technologies Center (AMTC), National Research Council Canada, Montreal, QC H3T 1J4, Canada; 3 Department of Mechanical Engineering, Polytechnique Montréal, Montreal, QC H3T 1J4, Canada; farbod.khameneifar@polymtl.ca

**Keywords:** damaged blade inspection and remanufacturing, denoising and outlier removal, simplification, efficiency, scan-to-CAD rigid registration, CAD-to-scan non-rigid registration

## Abstract

Point cloud processing of raw scan data is a critical step to enhance the accuracy and efficiency in computer-aided inspection and remanufacturing of damaged aero-engine blades. This paper presents a new methodology to obtain a noise-reduced and simplified dataset from the raw scan data while preserving the underlying geometry of the damaged blade in high-curvature and damaged regions. At first, outliers are removed from the scan data, and measurement noise is reduced through local least-squares quadric surface/plane fitting on the adaptive support domain of measured points under the measurement uncertainty constraint of inspection data. Then, a directed Hausdorff distance-based region growing scheme is developed to progressively search within the support domain of denoised data points to obtain a down-sampled dataset while preserving the local geometric shape of the surface. Numerical and experimental case studies have been conducted to evaluate the accuracy and computation time of scan-to-CAD rigid registration and CAD-to-scan non-rigid registration processes using the down-sampled dataset of damaged blades. The results have demonstrated that the proposed methodology effectively removes the measurement noise and outliers and provides a down-sampled dataset from the scan data that can significantly reduce the time complexity of the computer-aided inspection and remanufacturing process of the point cloud of damaged blades with a negligible loss of accuracy.

## 1. Introduction

**Figure 1:**
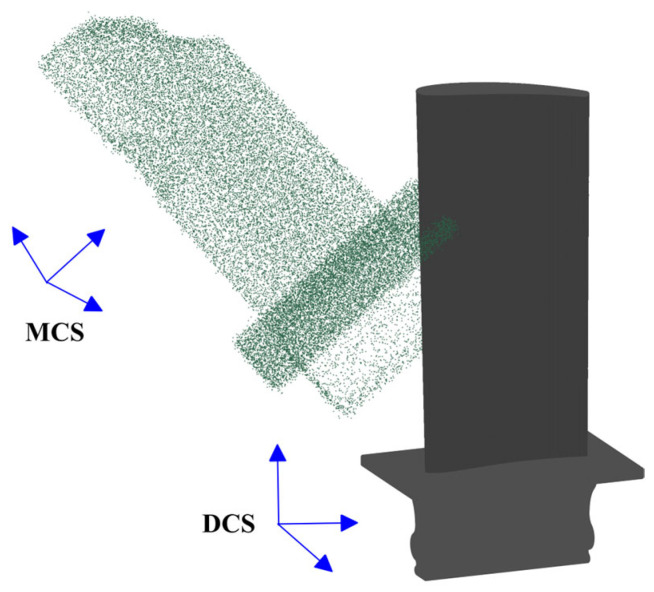
The raw scan data of a damaged blade and its initial position and orientation relative to the CAD model.

**Figure 2:**
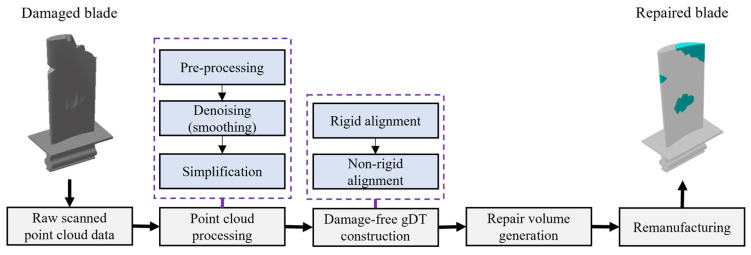
Outline of 3D scanning-based inspection and repair of damaged blades.

With the development of modern industrial manufacturing, 3D scanning technologies (e.g., structured light scanners and laser scanners) can accurately capture hundreds of thousands of data points from the part’s surface in just a few seconds [1]. In the maintenance, repair, and overhaul (MRO) industry, one of the primary applications of 3D scan data is computer-aided inspection (CAI) of aero-engine blades to check for their conformance to the specified tolerances over time [2]. Typically, deviations of in-service and damaged blades from their original geometric shape are evaluated through part-specific and section-specific inspection of the captured 3D point cloud [3,4]. In the case of damaged blades, repairing the material-missing regions—rather than replacing the entire component—can often be a more cost-effective solution to extend their service life [5,6]. Figure 1 shows the raw 3D scan data of a damaged blade as well as the CAD model. As illustrated in Figure 1, the scanned point cloud data is contaminated by measurement noise and outliers and lies in the measurement coordinate system (MCS), which has an arbitrary relative position and orientation with respect to the design coordinate system (DCS) in which the nominal CAD model lies. Figure 2 illustrates the outline of 3D scanning-based computer-aided inspection (CAI) and repair for damaged aero-engine blades in the presence of the nominal CAD model. The process begins with acquiring the raw point cloud of the damaged blade using 3D scanning, followed by point cloud processing to improve the data quality for the subsequent stages. The core of the framework focuses on constructing the damage-free geometric digital twin (gDT) of the 3D scanned defective blade and yielding the repair volume in material-missing regions. To construct the damage-free gDT, it is required to perform two main processes on data: scan-to-CAD rigid registration and CAD-to-scan non-rigid registration [4,7]. The scan-to-CAD rigid registration brings the scan data to a common coordinate system with the CAD model [8,9]. Then, the CAD-to-scan non-rigid registration is employed to gradually deform the CAD model dataset in deformed regions to match it with the measured data and generate the damage-free gDT of the damaged blade. Finally, the repair volume in material-missing areas is obtained through a Boolean operation between scan data and the damage-free gDT [7].

The accuracy and efficiency of the rigid and non-rigid registration algorithms are directly affected by the quality and density of the measured point cloud used for inspection and repair. The raw scan data of the damaged blade is a dense point cloud containing measurement noise and outliers on the surface, which can be especially more pronounced in high curvature regions (i.e., the leading and trailing edges). The presence of measurement noises and outliers on the scan data can adversely affect the correspondence search in the rigid registration algorithm [8,10]. Mainly, the averaging-out errors can be intensified by outliers in damaged regions and damage boundaries. In the non-rigid registration, the presence of measurement noise and outliers will introduce unreliable correspondences between CAD points and scan data, resulting in shrinkage and expansion of data points of the constructed damage-free gDT. Consequently, the Boolean operation between scan data and the damage-free gDT of the damaged blade may yield an inaccurate representation of repair volume, with unsmooth continuity between repaired and unrepaired regions [7]. To avoid the noted inaccuracies in the rigid and non-rigid alignment of scan data, there is a definite need to eliminate the measurement noise and outliers from the raw scan data of damaged aero-engine blades.

In addition to accuracy, computational efficiency is an essential factor in the effectiveness of computer-aided inspection (CAI) of 3D scanned point clouds. Applying a dense point cloud of the damaged blade for rigid and non-rigid alignment processes can dramatically increase the computation time and memory footprints. The correspondence search and transformation matrix calculation are two parameters affecting the run time. In each iteration of rigid and non-rigid registrations, the algorithm should establish the correspondence between measured data points and the CAD model. Therefore, it takes a long time to perform the correspondence search for dense point cloud data of the damaged blade, especially when the correspondence search method differs from the common closest point-to-point approach (e.g., group-to-group correspondence search [8] and point-to-surface correspondence search [7]). Also, the running time for the transformation matrix calculation of dense point clouds is longer in rigid and non-rigid registration processes. The most time-consuming step in the presented framework, in Figure 2, is the calculation of transformation matrices for CAD-to-scan non-rigid registration. In each iteration of the non-rigid registration, the algorithm should compute an individual transformation matrix for each corresponding pair to project deformations onto the CAD model, which dramatically increases the computation time. It should be noted that, in CAD-to-scan non-rigid registration, both datasets should have identical average point spacing [7]. Thus, by increasing the density of the scanned point cloud, the computational complexity of damage-free gDT construction increases. To overcome the mentioned computational complexities of CAI of the damaged blade and damage-free gDT generation, it is necessary to simplify (down-sample) the measured point cloud of the damaged aero-engine blade while preserving the underlying local geometry.

The motivation of this paper is to propose a point cloud processing approach in order to improve the accuracy and efficiency in 3D scanning-based computer-aided inspection and repair volume generation of damaged blades. As illustrated in Figure 2, the proposed methodology aims to denoise and simplify the raw scan data of a damaged blade (with material-missing regions and considerable deformations on the surface) to provide a clean and down-sampled dataset for scan-to-CAD rigid registration and CAD-to-scan non-rigid registration processes. This study develops a workflow that combines uncertainty-driven denoising, outlier removal, adaptive local fitting, and progressive down-sampling to improve rigid and nonrigid registration accuracy and efficiency for damaged blades to construct damage-free gDT. The point cloud processing approach includes three main steps: (1) pre-processing, (2) denoising, and (3) simplification. The main features of each step are outlined as follows:

- Pre-processing: pre-processing aims to establish the local neighborhood (neighboring points) and normal direction of each data point of the raw 3D scanned point cloud and identify the type of local underlying geometry (i.e., planar or quadric surface) through principal component analysis (PCA).
- Denoising: the purpose of the denoising process (also known as smoothing and filtering) is to automatically project the noisy data points onto the local underlying surface and eliminate the outliers from the scan data. A progressive weighted local least-squares method is devised to fit a planar or quadric surface on the adaptive local neighborhood of each measured data point under the measurement uncertainty constraint. The outliers are automatically detected and eliminated by analysis of projections of each scan data point within its expanded uncertainty interval.
- Simplification: the main idea behind simplification is to down-sample the smoothed scan data while preserving the original geometric features, mainly in high curvature regions, damage boundaries, and material missing areas. The algorithm benefits from a directed Hausdorff distance-based region-growing approach to search for the support domain of each smoothed data point and provide a uniform down-sampled point cloud of the damaged blade, containing as much of the local geometric shape of the surface as possible.

In this study, the validation of the proposed methodology for denoising and simplification is limited to raw scan data of damaged aero-engine blades, since they represent a high-priority case in MRO where remanufacturing is of significant economic and technical interest compared to full replacement. Moreover, the alignment evaluation in this paper relies on scan-to-CAD rigid registration [8] and CAD-to-scan non-rigid registration [7] algorithms, both of which have been primarily developed and tailored for damaged blade geometries.

## 2. Related Works

Point cloud smoothing and simplification are active research areas in computer graphics and computer vision with numerous applications. Denoising or smoothing, in computer-aided inspection of aero-engine blades, aims to recover the ideal point cloud by removing unwanted measurement noises and outliers from raw scan data to enhance the accuracy in part inspection. One of the main challenges in the denoising of raw scan data of the blade is that the leading and trailing edges, as high curvature features, tend to be more contaminated by measurement noises and outliers [11]. Using an inappropriate denoising algorithm can excessively smooth the point cloud, causing shrinkage in leading/trailing edges, or in suction and pressure sides [12,13]. Schall et al. [14] introduced a non-local filtering approach to denoise meshes using similar vertices in their adaptive neighborhood, based on a non-local similarity measure. This method is exclusively designed for denoising (not outlier removal). Local approximation for similarity evaluation in non-local denoising methods can lead to significant computational overhead. The bilateral method is one of the smoothing approaches utilized for denoising the raw scan data of blades [12,13,15]. It is a weighted Gaussian filter with a feature-preserving weighting function to smooth the point cloud based on position, normal, color, etc. [16]. Wang et al. [15] applied the point cloud bilateral filter algorithm to reduce the measurement noise of the aero-engine blade. Li et al. [12,13] proposed an adaptive bilateral smoothing method for denoising the raw scan data of blades, utilizing the information entropy theory to differentiate the density variations within the point cloud and select the optimal parameters for smoothing the surface while preserving thin-walled features (i.e., trailing and leading edges). Zheng et al. [17] proposed an algorithm to divide the scan data points into outliers, feature-rich, and flat regions. The algorithm removed outliers directly and smoothed other data points via the bilateral filtering method and weighted average filtering approach [17]. Although the bilateral filter method is simple and non-iterative, its accuracy is lower than that of least-square fitting in dealing with sharp features (high curvature regions) like leading and trailing edges of blades [11].

In addition to denoising the point cloud, outliers should be detected and removed from the raw scan data. Depending upon the correlation among the outliers, we can have sparse, isolated, or non-isolated outliers in the scan data [18,19]. In this study, the focus is on removing sparse outliers with low local density from scanned damaged blades. In general, the sparse outliers are detected by analyzing the condition of each measured data point relative to its local neighborhood. There are various outlier removal approaches, such as distance-based methods [20,21,22], local plane fitting [21], statistical methods [23], local density analysis [18,24,25], majority voting method [19], etc. One of the main challenges in existing methods for denoising and outlier removal of measured scan data is properly assigning user-defined thresholds to obtain reliable results. Since automation plays a crucial role in computer-aided inspection of damaged blades, it is essential to avoid user-defined parameters and thresholds as much as possible. In this study, we adopt the measurement uncertainty of the inspection data points as a consistent criterion to automatically denoise the raw scan data and detect the outliers.

To enhance the efficiency of downstream processes on the smoothed point cloud of the damaged blade, it is necessary to down-sample the scan data while preserving the underlying geometry of the point cloud, particularly in high-curvature regions, such as the leading edge, trailing edge, and damage boundaries, as much as possible. Uniform, nonuniform [26], random [27], and curvature-based [28] simplification methods are widely used approaches for down-sampling scanned point clouds. Li et al. [13] proposed a clustering-based method for feature-preserving simplification of the point cloud of the blade. They applied the moving least square surface fitting to *k*-nearest neighbors (*k*-NNs) of each point to identify the geometric deviation of the point from its local surface for subdivision and clustering the point cloud. Li et al. [29] developed a mixed sampling method in which *k*-means clustering and the directed Hausdorff distance technique are combined to down-sample the point cloud data. In another research, Li et al. proposed an enhanced max-min distance method [30] to simplify the scan data for registration and inspection of newly manufactured blades. The methods proposed in [13,29,30] require several user-defined parameters and thresholds for the simplification process. Also, these algorithms employ *k*-nearest neighbors to search the point cloud for simplified points, which does not guarantee preserving the underlying geometry of scan data, especially in cases of uneven distribution of points. Zhang et al. [31] presented a feature-preserved point cloud simplification methodology in which the local geometric feature around each key point is specified based on simplification entropies, and then, using designed simplification rules, the neighbors of key points are down-sampled. The simplification rules and entropies designed in [31] are not applicable to free-form features like blade surfaces and require several user-defined thresholds for implementation.

Table 1 summarizes representative neighborhood-based point cloud denoising and simplification methods that are commonly reported in the literature and considered relevant for aeroengine blade applications. Bilateral filtering and its variants are primarily employed to denoise raw scan data by considering both spatial proximity and normal similarity through spatial and range weights. Clustering- and entropy-based simplification methods address downsampling by grouping or prioritizing points according to their geometric significance (e.g., edges, high-curvature zones). The table also highlights comments regarding the advantages and drawbacks of each technique, showing that while these methods offer simplicity and efficiency, they typically rely on multiple user-defined parameters and thresholds, which prevent them from being fully automated. Furthermore, these approaches can be computationally expensive and may risk over-smoothing or over-simplifying the scan data. This study aims to develop an automated and efficient methodology for smoothing and simplifying the raw scan data of damaged blades, while preserving the underlying geometry under the constraint of measurement uncertainty.

In this work, we apply an adaptive support domain obtained by local surface fitting under the measurement uncertainty constraint to search for simplified data points in the local neighborhood of points. In addition, a Hausdorff distance-based region growing methodology is developed in this work to progressively search for simplified points within each adaptive support domain and generate a subset of scan data. Numerical and experimental case studies have been conducted to evaluate the performance of the scan-to-CAD rigid registration and CAD-to-scan non-rigid registration processes in aligning the simplified datasets of synthetic and scanned point clouds of damaged blades.

## 3. Proposed Methodology

### 3.1. Pre-Processing

The aim of data pre-processing is to obtain some useful information on the local underlying geometry of the raw scanned point cloud data, which will be used in the subsequent steps for denoising and simplification. Algorithm 1 describes the pre-processing of the raw scan data of the damaged blade. First, the neighboring points of each original point ($p_{i}$) are established by the territory claiming (TC) algorithm [34] to obtain the neighboring points NB($p_{i}$) around the center point $p_{i}$. TC algorithm employs a distance-based criterion to find the closest directionally balanced set of neighboring points. In this algorithm, rings of points are proposed to maintain balance when the neighborhood needs to grow to include more points [34]. We obtain local neighboring points for each measured data point using the TC algorithm. Then, Principal Component Analysis (PCA) is performed on 10 local neighboring points to roughly approximate the type of underlying surface (local feature) at the point $p_{i}$ as a planar or non-planar feature. PCA is a statistical technique to explain the covariance structure of the data using its principal components [35]. For each point $p_{i}$, the corresponding orthogonal directions are given by eigenvectors of the covariance matrix $M_{c}$ of neighboring points NB($p_{i}$):

(1) $$M_{c}(p_{i})=\sum_{j=1}^{NB(p_{i})}(p_{j}-O)(p_{j}-O{)}^{T}$$

where $p_{j}$ is the *j*th point of dataset *NB*($p_{i}$) and *O* is the centroid of the dataset *NB*($p_{i}$). The surface variation ${sv(p}_{i})$ at point $p_{i}$ is defined as [36]:

(2) $${sv(p}_{i})=\frac{\lambda_{0}}{\lambda_{0}+\lambda_{1}+\lambda_{2}}$$

where $\lambda_{l}\;\left(l=0,1,2\right)$ is the eigenvalue $\left(\lambda_{0}\leq \lambda_{1}\leq \lambda_{2}\right)$. The ${sv(p}_{i})$ quantitatively describes how much local neighboring data points of $p_{i}$ deviate from the tangent plane. We define a threshold value δ in which for ${sv(p}_{i})$ larger than the δ local neighborhood is considered as quadric surface and for ${sv(p}_{i})$ smaller than δ the local neighborhood is a planar surface. In this study, the value of δ is equal to 0.0005, which is obtained by trial and error for different datasets. Finally, the normal vector $v_{i}$ at each scan data point is obtained by fitting a plane or a quadric surface to neighboring points *NB*($p_{i}$). This information will be employed in the proposed methodology to remove the outliers and smooth the raw scanned point cloud of the damaged blade in the next step.

**Algorithm 1** Data pre-processing**Input:** raw scan data P={$p_{0},\;p_{1},\ldots ,\;p_{n}$} and surface variation threshold δ. **Output:** local neighborhood and local properties of scan data. 1. **for**
*i* = 0 to *n* **do** 2. Establish neighboring points *NB*($p_{i}$) around the point $p_{i}$. 3. Principal component analysis of neighboring data points *NB*($p_{i}$) to get eigenvalues ($\lambda_{0}$, $\lambda_{1}$, $\lambda_{2}$) and eigenvectors ($v_{0},v_{1},v_{2}$). 4. Compute the surface variation $sv\left(p_{i}\right)$ using Equation (2) 5.    **if**
$sv\left(p_{i}\right)<\delta$ **then** 6.      Local neighborhood of point $p_{i}$ is planar 7.      Fit a least-square plane on neighboring points *NB*($p_{i}$) to get normal vector $\vec{N_{p_{i}}}$ 8.      **else** 9.      Local neighborhood of point $p_{i}$ is non-planar 10.    Fit a least-square quadric surface on neighboring points *NB*($p_{i}$) to get normal vector $\vec{N_{p_{i}}}$ 11.  end if 12. end for 13. **Return**: local neighborhood set ${NB}_{P}=\{NB\left(p_{0}\right),NB\left(p_{1}\right),\ldots ,NB(p_{n})\;\}$, surface variation set ${SV}_{P}=\{sv\left(p_{0}\right),sv\left(p_{1}\right),\ldots ,sv\left(p_{n}\right)\}$, and normal vector set $N_{P}=\{\vec{N_{p_{0}}},\vec{N_{p_{1}}},\ldots ,\vec{N_{p_{n}}}\;\}$.

### 3.2. Smoothing

This study introduces an enhanced method using weighted local least squares to fit a plane or a polynomial surface (based on surface variation value) to the neighboring points *NB*($p_{i}$) of each measured point $p_{i}$ to approximate the local underlying geometry and denoise the point $p_{i}$ within its measurement uncertainty interval. Algorithm 2 describes the proposed point cloud smoothing approach. We employ a weighting function to measure the closeness of each point to its local neighborhood and eliminate the influence of measurement noise and outliers on the local surface fitting procedure. The proposed weighted local least squares method benefits from an adaptive support domain to progressively cover more neighboring data points and obtain a fitted plane or surface with high conformity to the underlying geometry. That is, the weighted local least squares fitting process starts with the minimum possible neighboring points (depending on the type of local feature, i.e., plane or surface, and the degree of fitted polynomial) and then the number of neighboring points gradually increases to expand the support domain and estimate the underlying geometry with more data points. The fitting procedure is terminated when the fitted residuals meet the specified stopping criterion. The main steps of the smoothing process are weighting of measured data points, weighted local least squares fitting, and outlier removal.

**Algorithm 2** Smoothing**Input:** raw scan data P, local neighborhood set ${NB}_{P}$, surface variation set ${SV}_{P}$, normal vector set $N_{P}$, and measurement uncertainty *u*. **Output:** a smooth (denoised) point cloud dataset of the scanned damaged blade 1. **for**
*j* = 0 to *n* **do** 2. Compute the local outlier factor for each measured data point $p_{j}$ using Equations (3)–(6). 3. end for 4. Calculate the standard deviation of outlier factor values of data points: $\sigma_{out}$. 5. **for** *j* = 0 to *n* **do** 6. Compute the local outlier weight value for each data point $p_{j}$ using Equations (5)–(6). 7. end for 8. **for**
*i* = 0 to *n* **do** 9.**    if** $sv\left(p_{i}\right)\leq \delta$ **then** (planar local neighborhood) 10.  ${SupD(p}_{i})\leftarrow \;$pick the number of rings containing at least 3 nearest neighboring points $NB(p_{i})$ for plane fitting 11.    **while** RMSE≤u **do** 12.    WLLS plane approximation on adaptive support domain ${SupD(p}_{i})$ and obtain the *RMSE* of residuals. 13.     ${SupD(p}_{i})\leftarrow {SupD(p}_{i})\cup \;$one ring of neighboring points. 14.     end while 15.**  elseif**
$sv\left(p_{i}\right)>\delta$ **then** (quadric local neighborhood) 16.  ${SupD(p}_{i})\leftarrow$ pick the number of rings containing at least 6 nearest neighboring points $NB(p_{i})$ for quadric surface fitting 17.     **while** RMSE≤u **do** 18.     WLLS quadric surface approximation on adaptive support domain ${SupD(p}_{i})$ and obtain the *RMSE* of residuals. 19.     ${SupD(p}_{i})\leftarrow {SupD(p}_{i})\cup \;$one ring of neighboring points. 20.     end while 21.     $Pro({SupD(p}_{i}))\leftarrow$ projection of data points in the support domain ${SupD(p}_{i})$ 22.  end if 23. end for 24. **for**
*i* = 0 to *n* **do** 25. Find the data points whose support domain includes the point $p_{i}$ and obtain the projections of  $p_{i}$ 26. Compute the mean of the projections of $p_{i}$: $s_{i}$ 27.   **if** $\|s_{i}-p_{i}\|\leq 3u$ **then** 28. ${P_{D}\leftarrow s}_{i}$ 29. **elseif** $\|s_{i}-p_{i}\|>3u$ **then** 30. $p_{i}$ is an outlier. 31. end 32. **Return**: the denoised dataset $P_{D}=${$s_{0},\;s_{1},\ldots ,\;s_{m}$}.

#### 3.2.1. Weighting

The presence of measurement noise and outliers may adversely affect the local least squares surface (or plane) fitting and deviate the fitted surface from the original underlying geometry. We use a weighting function to assign a weight factor to each query data point $p_{i}$ based on the distance of the point to its local neighboring points *NB*($p_{i}$) [3,22]. For each measured data point $p_{i}$, the neighborhood distance $\bar{d}\left(p_{i}\right)$ is defined as the average Euclidean distance of $p_{i}$ to the point $q_{j}$ in its local neighborhood *NB*($p_{i}$) [22]:

(3) $$\bar{d}\left(p_{i}\right)=\frac{1}{\left|NB\left(p_{i}\right)\right|}\sum_{q_{j}\;\in \;NB\left(p_{i}\right)}\left\|p_{i}-q_{j}\right\|$$

Also, the neighborhood inner distance $\bar{D}\left(p_{i}\right)$ is calculated as the average distance between any two points ($q_{j}$ and $q_{k}$, *j* ≠ *k*) within the local neighborhood points of $p_{i}$ ($NB\left(p_{i}\right)$):

(4) $$\bar{D}\left(p_{i}\right)=\frac{1}{\left|NB\left(p_{i}\right)\right|\left(\left|NB\left(p_{i}\right)\right|-1\right)}\sum_{q_{j},\;q_{k}\;\in \;NB\left(p_{i}\right),\;j\neq k}\left\|q_{j}-q_{k}\right\|$$

The local distance-based outlier factor $OF\left(p_{i}\right)$ value and the weighting function $w\left(p_{i}\right)$ for the point $p_{i}$ are computed, respectively, using Equations (5) and (6) [22]:

(5) $$OF\left(p_{i}\right)=\frac{\bar{d}\left(p_{i}\right)}{\bar{D}\left(p_{i}\right)}$$

(6) $$w\left(p_{i}\right)=exp\left(\frac{-OF\left(p_{i}\right)\;}{\sigma_{out}}\right)$$

The $OF\left(p_{i}\right)$ roughly indicates how far the point $p_{i}$ lies outside its local underlying geometry. For example, if most points in a neighborhood form a smooth surface patch but one point lies significantly above the fitted surface (e.g., due to scanning noise or reflection), its outlier factor will be much larger than that of the surrounding points, giving it a higher chance of being classified as an outlier. Thus, the data point of larger $OF\left(p_{i}\right)$ is more deviated from its local geometry and is more of an outlier. The exponential weighting function $w\left(p_{i}\right)$, also, is introduced to give weight to each measured point based on its deviation from the local underlying geometry [3]. $\sigma_{out}$ is the standard deviation of outlier factor values of all points of the dataset P. Employing this weighting function, the local surface/plane fitting results will not be affected by outliers within the support domain.

#### 3.2.2. Weighted Local Least Squares Approximation

After assigning weight to data points, based on the local feature of each measured point $p_{i}$, we fit either a plane $\hat{f}\left(x,y\right)=\;c_{1}+c_{2}x+c_{3}y$ (${sv}_{i}\left(p_{i}\right)\leq \delta$) or a second-degree bivariate polynomial surface $\hat{f}\left(x,y\right)=\;c_{1}+c_{2}x+c_{3}y+c_{4}x^{2}+c_{5}xy+c_{6}y^{2}$ (${sv}_{i}\left(p_{i}\right)>\delta$) to the support domain ${SupD(p}_{i})$ containing *K* neighboring points $\{q_{k}=\left(x_{k},y_{k},z_{k}\right)|k=1,\ldots ,K\}\in S$. The plane and polynomial surface fitting is conducted using a weighted local least-squares (WLLS) fitting method, in which the algorithm finds the coefficients $c_{i}$ (*i* = 1,2,3,4,5,6) that minimize the summation of weighted residuals using the loss function [3]:

(7) $$J=\sum_{k=1}^{K}{\left(\hat{f}\left(x_{k},y_{k}\right)-z_{k}\right)}^{2}w(q_{k})$$

It should be noted that the weight $w(q_{k})$ of each neighboring point is already computed by Equation (6) in the previous step. Also, the neighboring points are transformed to align the normal direction at the query point $p_{i}$ with the *z* direction of the surface fitting coordinate system. An adaptive support domain ${SupD(p}_{i})$ is utilized for the least-squares fitting procedure in order to progressively comprise more neighboring points and obtain a fitted plane or surface with high conformity to the underlying geometry. In this study, the adaptive support domain is expanded by adding rings of neighboring points to cover the weighted least squares approximation in all directions. The algorithm begins with the smallest fitting support domain in which we pick the number of rings containing the minimum possible nearest neighbors, i.e., at least 3 points for plane fitting and at least 6 points for second-degree bivariate polynomial surface fitting. Then, by extension of the support domain ${SupD(p}_{i})$ (adding a new ring of neighboring points), the least squares estimator is updated to minimize the objective function in Equation (7) and obtain the residuals. Due to the fact that the raw scanned point cloud data is subject to measurement uncertainty, the actual point of each measured data is somewhere within a sphere centered at the measured point with the radius equal to the expanded uncertainty 3*u* where *u* is the standard uncertainty of the point cloud data [37]. Although measurement uncertainty can vary with device specifications, calibration state, and scanning environment, employing this parameter enhances the performance of the algorithm by relying on an automated statistical criterion rather than a subjective user-defined threshold. Since the value of measurement uncertainty is given, we utilize this parameter to define the stopping threshold of local least squares approximation. In this work, the algorithm terminates extending the support domain ${SupD(p}_{i})$ for weighted local least squares fitting when the root mean square error (RMSE) of the fitted residuals of neighboring data points within the support domain gets larger than standard uncertainty *u* of the point cloud:

(8) RMSE>u

By employing the standard uncertainty as the stopping threshold, the algorithm statistically distinguishes between measurement noise and underlying geometry around each point. This ensures that deviations consistent with noise are smoothed out and geometric details are preserved, which is critical for reliable denoising and outlier removal. Since in each iteration, the algorithm adds a ring of neighboring points to extend the support domain with the constraint of standard uncertainty of the data points, the number of data points utilized for least squares approximation is not fixed and varies for each measured data point depending on the noise and outlier distribution, point cloud density, and underlying geometric shape in the vicinity of that point.

#### 3.2.3. Data Projection and Outlier Removal

**Figure 3:**
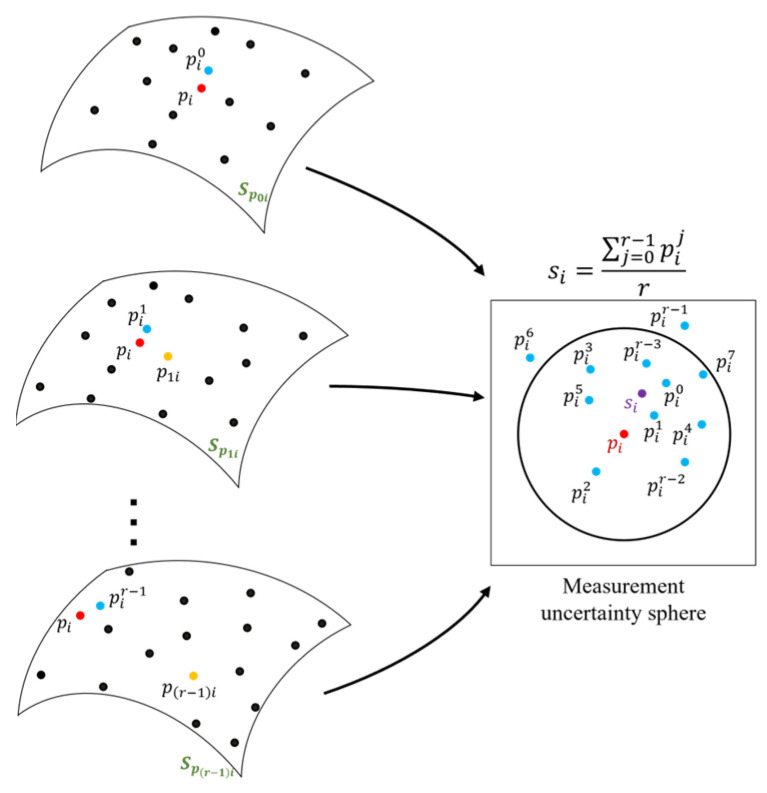
Projected coordinates of point $p_{i}$ after local surface fitting on the local neighborhoods of *r* measured data points whose adaptive support domain includes the point $p_{i}$. The point $s_{i}$ is the mean of projected coordinates of point $p_{i}$. As can be seen, the point $s_{i}$ is within the measurement uncertainty sphere and is selected as a smooth data point (not an outlier).

When we fit a plane or polynomial surface to the adaptive support domain of each measured data point $p_{i}$ using the weighted local least squares method, data points within the support domain are projected onto the fitted surface in the normal direction of the point $p_{i}$. In order to guarantee the smoothness between projected data points of all locally fitted planes and polynomial surfaces, it is required to make the connection between locally fitted surfaces. To this end, we generate a map of projections for each measured data point $p_{i}$. In this procedure, all locally fitted planes and polynomial surfaces containing the query point $p_{i}$ within their support domain are determined and the projected coordinates of the point $p_{i}$ are obtained. We suppose that each measured point $p_{i}$ could be included in the support domain of *r* measured data points. Thus, as can be seen in Figure 3, for each measured point $p_{i}$, there are *r* projected coordinates $R_{p_{i}}=\{p_{i}^{0},\;p_{i}^{1},\;p_{i}^{2},\ldots ,\;p_{i}^{j},\ldots ,\;p_{i}^{r-1}\;\}$. The projected coordinates $R_{p_{i}}$ are obtained via weighted local least squares approximation on the support domain of measured data points $Q_{p_{i}}=\{\;p_{0i},\;p_{1i},\ldots ,p_{ji},\ldots ,\;p_{(r-1)i}\}$ $\left(p_{ji}=\{p\;|\;p\in P,\;p_{i}\in {SupD(p}_{j})\right)$. As mentioned earlier, the actual point of each measured data point is somewhere within a sphere centered at the measured point with the radius equal to the expanded uncertainty 3*u*. Therefore, the projected data points $R_{p_{i}}$ are mapped into the uncertainty sphere centered at the point $p_{i}$, as shown in Figure 3. In this study, for each measured data point $p_{i}$, the filtered data point $s_{i}$ is defined as the mean value of projected coordinates at dataset $R_{p_{i}}$:

(9) $$s_{i}=\frac{1}{r}\sum_{j=0}^{r-1}\left(p_{i}^{j}\right)$$

To ensure feasible smoothing results, the smoothed (denoised) data point $s_{i}$ should be in the uncertainty sphere of the measured point $p_{i}$. If the smoothed data point is out of the uncertainty sphere of its original measured point, it means that the measured point is significantly far from its local underlying geometry. In this study, the measured data points whose filtered (smoothed) corresponding point is out of their measurement uncertainty sphere are considered outliers. Therefore, the smoothed data points whose distance from the original measured point is larger than 3*u* ($\|s_{i}-p_{i}\|>3u$) are removed from smoothed dataset as outliers. As a result of averaging of projections and outlier removal, a denoised and smoothed dataset $P_{D}=\{\;s_{1},\;s_{2},\ldots ,\;s_{m}\}$ of the raw scanned dataset *P* is obtained. Employing an adaptive support domain for least-squares fitting, together with constructing the map of projections for each measured point, reduces the risk of propagating local fitting errors into the global simplification result. In the next step of our proposed method, the smooth dataset $P_{D}$ serves as the input for point cloud data simplification.

### 3.3. Simplification

Once the raw scanned point cloud is filtered and denoised, the simplification process is performed to downsample the smoothed dataset in order to improve the efficiency of rigid scan-to-CAD and non-rigid CAD-to-scan registrations. A reliable simplification approach should effectively reduce the density of point cloud data while preserving the underlying geometry of the scan data, particularly in high curvature regions and defective areas of the damaged blade. Thus, it is required to down-sample the denoised point cloud based on the local geometric variations in the surface. In this work, we propose a new method to progressively select data points as far apart as possible within their support domain, while effectively preserving the local geometric shape of the smoothed point cloud. Algorithm 3 describes the proposed simplification methodology. A region-growing-based technique is employed for simplifying the point cloud, in which the algorithm starts with the support domain of the denoised point of interest, referred to here as the simplification domain, and grows the set of simplified data points until it provides a simplified point cloud of the entire damaged blade. A directed Hausdorff distance-based approach is applied to find the best candidate as a simplified data point via search in the simplification domain of each denoised data point $s_{i}$. It should be noted that the support domain of the smoothed data points (i.e., simplification domain) is identical to the support domain of the original scanned data points adopted for local weighted least squares approximation. Since the support domain of data points represents the local underlying geometry around them under the measurement uncertainty constraint, simplification of the point cloud through downsampling the support domain of data points guarantees to preserve the underlying geometry of the point cloud as much as possible.

**Algorithm 3** Simplification algorithm**Input:** denoised data points $P_{D}$ ={$s_{0},\;s_{1},\ldots ,\;s_{m}$} and their support domain ${SUP}_{D}=\{{SupD(s}_{0}),\ldots ,{SupD(s}_{m})\}$. **Output:** a downsampled dataset from the denoised dataset $P_{D}$. 1. Select the nearest denoised point $s_{0}^{\prime }$ to the centroid of the denoised dataset $P_{D}$ as the first simplified point: $P_{s}\leftarrow s_{0}^{\prime }$. 2. Select the farthest data point $s_{1}^{\prime }$ to the point $s_{0}^{\prime }$ within the support domain $SupD(s_{0}^{\prime })$ as the second simplified data point: $P_{s}\leftarrow P_{s}\cup s_{1}^{\prime }$. 3. Initial k=1;j=0; and reserve dataset: ${Res}_{P_{D}}=P_{D}$. 4. **while** ${Res}_{P_{D}}$ is not empty **do** 5.    Compute active and inactive data points in the support domain $s_{j}^{\prime }$: $Inact(s_{j}^{\prime })=SupD(s_{j}^{\prime })\cap \sum_{i=0\;s_{i}^{\prime }\in P_{s},i\neq j\;}^{k}SupD(s_{i}^{\prime })$, and $Act(s_{j}^{\prime })=SupD(s_{j}^{\prime })-Inact(s_{j}^{\prime })$. 6.    **while** $Act(s_{j}^{\prime })$ is not empty **do** 7.      k=k+1. 8.      Find the simplified data point $s_{k}^{\prime }\in Act(s_{j}^{\prime })$ using the directed Hausdorff distance approach (Equation (10)). 9.      Update the active data points in $SupD(s_{j}^{\prime })$: $I=Act(s_{j}^{\prime })\cap SupD(s_{k}^{\prime })$, $Act(s_{j}^{\prime })=Act(s_{j}^{\prime })-I$. 10.     $P_{s}\leftarrow P_{s}\cup s_{k}^{\prime }.$ 11.  end while 12.  ${Res}_{P_{D}}={Res}_{P_{D}}-SupD(s_{j}^{\prime })$. 13.  j=j+1. 14. end while 15. **Return:** simplified dataset $P_{s}=${$s_{0}^{\prime },\;s_{1}^{\prime },\ldots ,\;s_{t}^{\prime }$}.

**Figure 4:**
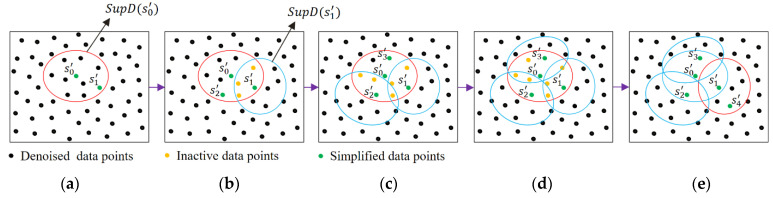
Simplification of the smoothed data points using a region growing-based technique to search within the support domain of each denoised data point $s_{0}^{\prime }$ for simplified data points: (**a**) starts with the support domain $supD(s_{0}^{\prime })$, (**b**) data points are divided into active points (black filled circles) and inactive points (orange filled circles) and the simplified data point $s_{2}^{\prime }$ is selected using Hausdorff distance criterion, (**c**–**e**) the algorithm searches among the updated active points to find simplified points $s_{3}^{\prime }$ and $s_{4}^{\prime }$.

In the simplification algorithm, the first and second simplified data points are initially defined as follows: the first simplified point $s_{0}^{\prime }$ is the nearest denoised point to the centroid of the denoised point cloud. The second simplified point $s_{1}^{\prime }$ is selected among the data points within the support domain $SupD(s_{0}^{\prime })$ which has the farthest distance to the point $s_{0}^{\prime }$. Once two initial simplified data points are specified and added to the simplified dataset $P_{s}$, the algorithm starts with the first data point $s_{0}^{\prime }$ at $P_{s}$ and assigns its support domain as the simplification domain to search for new simplified data points. In order to search for simplified data points in the simplification support domain of point $s_{j}^{\prime }$, the algorithm divides data points in the $SupD(s_{j}^{\prime })$ into two groups: inactive ($Inact(s_{j}^{\prime })$) and active ($Act(s_{j}^{\prime })$) data points. Inactive data points are the smoothed points within the simplification domain $SupD(s_{j}^{\prime })$ and the support domain of one of the previously selected simplified points, while the active data points only belong to the current simplification domain. The algorithm searches among the active data points within the simplification domain $Act(s_{j}^{\prime })$ to get the data point corresponding to the directed Hausdorff distance criterion as a new simplified point [29]:

(10) $$h\left(Act(s_{j}^{\prime }),P_{s}\right)=\underset{s_{i}\in \mathrm{Act}(s_{j}^{\prime })}{\max}\underset{s_{k}^{\prime }\in P_{s}}{\min}\left\|s_{i}-s_{k}^{\prime }\right\|$$

where $\left\|s_{i}-s_{k}^{\prime }\right\|$ is the Euclidean distance between two data points $s_{i}$ and $s_{k}^{\prime }$. The directed Hausdorff distance-based method is a sequential simplification approach in which all already selected simplified points affect the selection of new simplified points. In each iteration, the algorithm picks an active data point as far as possible from already selected simplified points in the dataset $P_{s}$ to gradually extend the simplified region. Figure 4 illustrates the simplification procedure in which two simplified data points $s_{0}^{\prime }$ and $s_{1}^{\prime }$ have been selected initially ($P_{s}=\{s_{0}^{\prime },s_{1}^{\prime }\}$). The algorithm starts with the support domain $supD(s_{0}^{\prime })$ to search for new simplified data point (Figure 4a). The data points within the $SupD(s_{0}^{\prime })$ are divided into active points $Act(s_{0}^{\prime })$ (black filled circles within the $SupD(s_{0}^{\prime })$) and inactive points $Inact(s_{0}^{\prime })$. As can be seen in Figure 4b, the inactive data points (orange filled circles) belong to the overlapping region of the $SupD(s_{0}^{\prime })$ and the support domain of the previously selected simplified data point $s_{1}^{\prime }$. By computing the directed Hausdorff distance between dataset $P_{s}$ and active data points, the data point $s_{2}^{\prime }$ is selected as a new simplified point and added to dataset $P_{s}$. Now, the active data points of $SupD(s_{0}^{\prime })$ belonging to the support domain $SupD(s_{2}^{\prime })$ are considered the inactive points and the algorithm searches among the updated active points to find new simplified points (Figure 4c). The algorithm terminates the search in the support domain of the point $s_{0}^{\prime }$ when there is no active point in the $SupD(s_{0}^{\prime })$ (Figure 4d). Then, the simplification algorithm searches within the support domain of the second simplified data point in $P_{s}$ to obtain new simplified points (Figure 4e). Using this procedure, the algorithm can locally search for new simplified data points by considering all already selected simplified data points. Therefore, by searching in the support domain of each data point of the denoised dataset, the algorithm not only provides a uniformly sampled dataset of points with a reasonable distance from each other but also the local underlying geometry details of scan data are preserved as much as possible. It is worth noting that since the number of active data points remains very small at each iteration, the computation cost of the Hausdorff distance remains reasonably low within the engineering-acceptable range, even when the simplified dataset becomes large.

## 4. Results and Discussion

**Figure 5:**
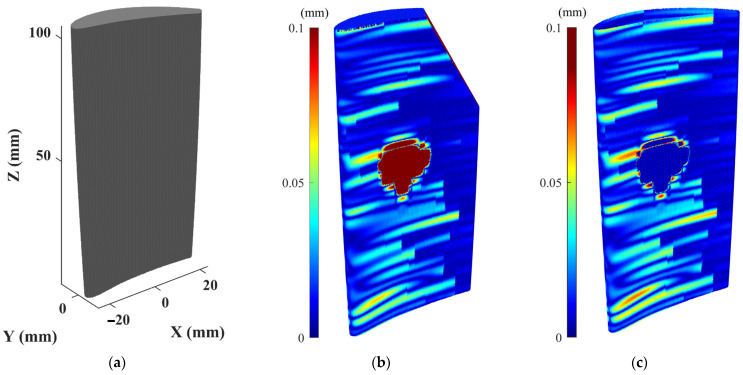
Numerical case study: (**a**) Nominal CAD model of the blade, (**b**) error colormap of the simulated point cloud (without noise and outlier) of the damaged blade, and (**c**) error colormap of the point cloud of the actual damage-free blade.

**Figure 6:**
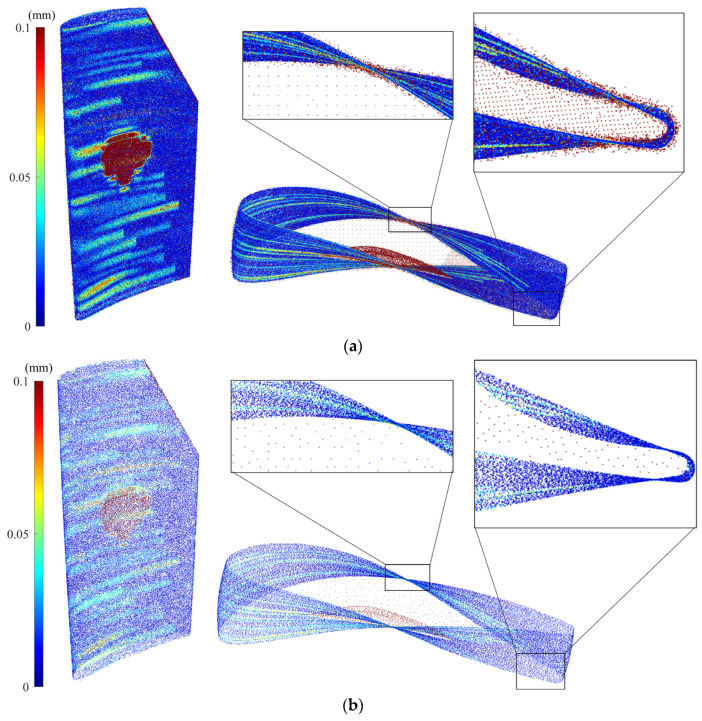
(**a**) Error colormaps of the synthetic raw point cloud (containing measurement noise and outliers) and (**b**) synthetic simplified point cloud of the damaged blade where measurement noises and outliers are effectively removed and a smooth and simplified dataset is obtained.

Numerical and experimental case studies have been conducted to validate the proposed data processing approach for denoising and simplification of the scanned point cloud data. For numerical case studies, synthetic point cloud data are generated by sampling a simulated damaged blade for which the actual geometry of the simulated blade is known. Thus, the actual geometry of the blade is utilized as the benchmark against which the accuracy of scan-to-CAD rigid registration and CAD-to-scan non-rigid registration outcomes can be compared to assess the performance of the proposed point cloud processing algorithms. The CAD model was created with the overall dimensions of the blade roughly corresponding to a cuboid of 45 mm in length, 15 mm in width, and 105 mm in height. To follow a typical blade surface design, a twist of 25 degrees from the blade bottom to tip was introduced by incrementally twisting the airfoil sections from the bottommost to the topmost section. To simulate the deformed blade surface, certain form deviations were superimposed onto the nominal blade surface. First, a sinusoidal variation with random amplitudes between 0 and 0.005 mm was superimposed onto the airfoil sections of the CAD model in the direction of the profile normal to emulate the combination of the typical manufacturing errors on the blade surface. Then, a sinusoidal variation with an amplitude between 0.04 and 0.08 mm was added to the pressure and suction sides of some airfoil sections of the blade to resemble a typical geometric deformation on a used blade during its operation. Next, geometric defects were added to the airfoil sections to simulate damages (material-missing regions) on the blade tip at the trailing edge as well as the pressure side of the blade surface. Using the NURBS surface interpolation of the airfoil sections, the simulated defective blade surface was created, which is then used as a reference. Figure 5 shows the nominal CAD model, as well as the simulated defective blade and simulated (actual) damage-free blade with their error colormaps with respect to the nominal CAD model. Since the maximum deformation of the actual damage-free blade (Figure 5c) with respect to the original CAD model is almost 0.1 mm, the error colormap range in Figure 5b is also capped at the same range (i.e., [0, 0.1] mm) for the sake of better visualization. Then, the simulated damaged blade surface was randomly sampled to generate an ideal (noise-free) point cloud. In practice, the scanned point cloud data contains measurement noise and outliers. To generate noisy point clouds of the simulated blade, Gaussian deviates with 0.01 mm standard deviation (with the distribution’s mean at zero) were superimposed onto the ideal point cloud in random directions. Finally, by superimposing the Gaussian deviates with 0.15 mm standard deviation (with the distribution’s mean at zero) onto 15% of the noisy point cloud in random directions to simulate the outliers on the point cloud, the synthetic point cloud of the damaged blade is generated. Figure 6a shows the synthetic raw point cloud of the damaged blade.

It is seen that there is considerable noise and outliers on the surface mainly in sharp regions. Using the methodology proposed in this paper, the synthetic point cloud of the damaged blade is denoised and simplified. Figure 6b presents the synthetic simplified model of the synthetic raw point cloud in which the outliers have been removed and a smooth and down-sampled model of the damaged blade is obtained. Table 2 indicates the number of data points of actual (without noise), synthetic raw (with noise and outliers), and synthetic simplified (smoothed and down-sampled) point clouds as well as the average deviation of point clouds from the CAD model when datasets are in the design coordinate system. The synthetic raw point cloud contains 379,750 points and the synthetic simplified dataset includes 46,681 points. It means that using the proposed point cloud processing approach, more than 87% of the data points are removed from the synthetic raw point cloud to obtain a denoised and simplified model of the damaged blade. In addition, the average deviation of the synthetic raw point cloud from the CAD model is 0.0556 mm while the deviation of actual and synthetic simplified point clouds from the CAD model, respectively, are 0.0391 and 0.0402 mm. The average error of the simplified point cloud nearly matches the average error of the actual point cloud of the damaged blade with only 0.0011 mm deviation. It shows that the proposed point cloud processing approach can effectively generate a smooth and down-sampled subset from the synthetic raw point cloud with a negligible loss of geometric detail (average error ≤ 0.0011 mm). It should be noted that, in general, the simplification of a point cloud may lead to the loss of some geometric detail in the underlying surface. Also, as can be seen in Table 2, computation times for denoising and simplification steps are, respectively, 44 and 53 s, which indicates the proposed methodology is relatively fast. For a comprehensive assessment of the effectiveness of the proposed methodology, it is necessary to conduct a systematic sensitivity analysis to evaluate the robustness of the proposed approach with respect to different input parameters, particularly measurement uncertainty and different types of measurement noise and outliers, which is beyond the scope of this study.

Since the raw scan data is not in a common coordinate system with the original CAD model, we introduce the initial misalignment to the synthetic raw and simplified point clouds by moving datasets to an arbitrary position and orientation with respect to the CAD model via a rigid body transformation. The initial misalignment, position ($X_{0},\;Y_{0},\;Z_{0}$) and orientation ($\theta_{x},\;\theta_{y},\;\theta_{z}$), of point clouds are set as ($X_{0},\;Y_{0},\;Z_{0}$) = (−25, −25, 25) mm and ($\theta_{x},\;\theta_{y},\;\theta_{z}$) = (0.1, −0.3, 0.3) radian.

### 4.1. Rigid and Non-Rigid Registration of Simulated Damaged Blade Point Clouds

In the case of a simulated damaged blade, we aim to align both synthetic and simplified point clouds of the damaged blade (Figure 6a,b) with the CAD model using the scan-to-CAD rigid registration method presented in [8,9]; and then, we generate the damage-free gDT of datasets through CAD-to-scan non-rigid registration methodology [7]. The registration results are compared for two datasets in terms of efficiency and accuracy. The scan-to-CAD rigid registration approach is employed to bring the synthetic raw and simplified point clouds of the simulated damaged blade into a common coordinate system with the original CAD model. The authors have recently developed a scan-to-CAD rigid registration algorithm [8], which includes three main steps: rough, fine, and fine-tuned alignments. The principal component analysis (PCA) technique is used for rough alignment to bring two datasets close to each other. For the fine alignment of scan data, the original iterative closest point (ICP) algorithm was employed, and then a fine-tuned registration process was utilized to iteratively eliminate the unreliable scanned data points of damaged regions from the registration process using a group-to-group dissimilarity evaluation of points in the local neighborhood to avoid the averaging-out issues of the original ICP method. The iteration in fine and fine-tuned registration algorithms is terminated when the change in registration global error falls below a specified threshold value [8].

The damage-free gDT of synthetic raw and simplified datasets is constructed using the CAD-to-scan non-rigid registration method developed by authors in Ref. [7]. Employing this method, after removing the data points of damaged regions, the CAD model is gradually deformed to match the dataset of the damaged blade in undamaged areas. In each iteration, the algorithm computes the transformation of each CAD data point to project the deformations of the damaged blade surface onto the CAD model while preserving the local rigidity as much as possible. It should be noted that the average point spacing of the point-sampled CAD model dataset is equal to that of scanned data. Thus, down-sampling the measured point cloud of the damaged blade directly affects the efficiency of nonrigid CAD-to-scan registration. Moreover, by employing a point-to-surface correspondence search scheme in CAD-to-scan nonrigid matching, the algorithm assigns a unique correspondence for each CAD data point on the local underlying geometry of its closest point on the simplified dataset. This approach avoids shrinking or expansion of the CAD model and minimizes the effect of any nonuniformity in the simplified data on the construction of damage-free gDT. For further details on rigid and non-rigid registration of damaged blades, readers are referred to Refs. [4,7,8,9].

The computations required for rigid scan-to-CAD alignment and non-rigid CAD-to-scan alignment were carried out in MATLAB R2022b on a PC with a 3.6 GHz Intel Core i7-7700 processor and 32 GB of RAM without parallel computing.

Table 3 indicates the average deviation of synthetic raw (Figure 6a) and synthetic simplified (Figure 6b) point clouds from the CAD model after scan-to-CAD rigid alignment, which are, respectively, 0.056 and 0.0412 mm.

According to the results in Table 2 and Table 3, the percent deviation of average error of synthetic and simplified point clouds from their ideal average values (in Table 2) is, respectively, 0.72% (0.0004 mm) and 2.49% (0.001 mm). That is, after rigid alignment, the average error percent deviation of the simplified point cloud is slightly higher than that of the synthetic raw dataset. This slight increase can be attributed to the marginal loss of geometric details during the point cloud simplification process. As shown in Table 3, the computation time for scan-to-CAD rigid registration is significantly reduced to 72 s for the synthetic simplified point cloud, in contrast to 769 s for the synthetic raw dataset, illustrating that the minor increase in error is a trade-off for significantly enhanced processing speed for the scan-to-CAD rigid registration. This marked improvement in computational efficiency arises because the simplified point cloud contains merely about 13% of the data points present in the synthetic model. The results demonstrated that the proposed simplification approach not only reduces the number of points in the cloud but also preserves essential geometric details, particularly in high-curvature and damage-boundary regions, thereby enabling faster registration without significantly compromising accuracy.

**Figure 7:**
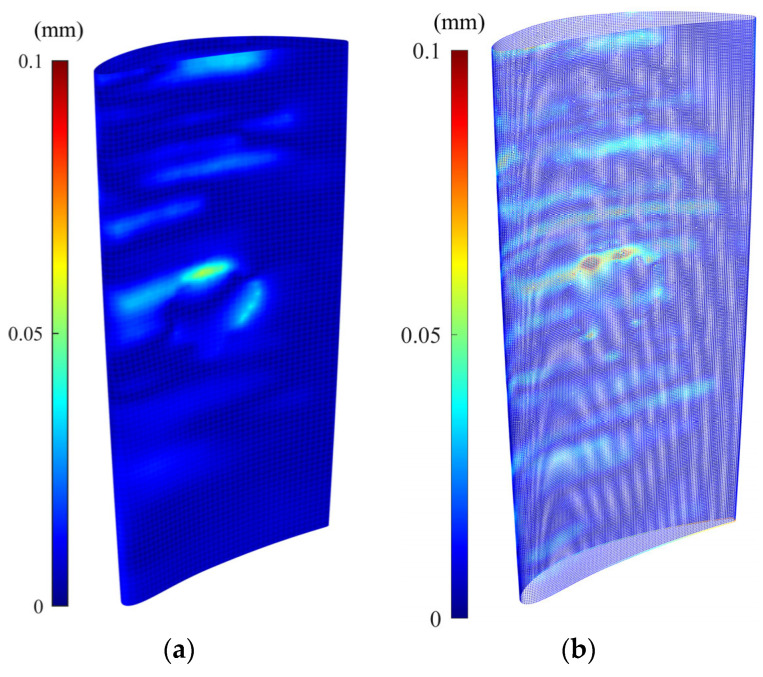
Constructed damage-free gDT of (**a**) synthetic point cloud and (**b**) simplified point cloud of the damaged blade using CAD-to-scan non-rigid registration method.

Figure 7 shows the error colormap (with respect to the CAD model) of the constructed damage-free gDT models for synthetic raw and simplified point clouds of the simulated damaged blade obtained through the CAD-to-scan non-rigid registration. Table 4 presents the average deviation of the actual damage-free gDT (Figure 5c) and constructed gDT models of the synthetic raw and simplified point clouds (Figure 7) from the original CAD model. As can be seen in Table 4, the average deviation of actual and simplified damage-free digital twins is 0.0145 mm and 0.0115 mm, respectively; that is, the percentage deviation of the average error between the two datasets is less than 21%. However, the average deviation of the synthetic damage-free digital twin from the CAD model is 0.0078 mm, indicating a percent deviation of more than 46% between the average errors of actual and synthetic damage-free digital twins. In order to guarantee the same average point spacing between two datasets in the CAD-to-scan non-rigid matching process, we have employed CAD models containing 1,144,710 and 101,332 points for damage-free digital twin construction of synthetic and simplified point clouds, respectively. It should be noted that CAD point clouds are generated by uniformly sampling the NURBS surface of the CAD model (with a specified average point spacing) while the synthetic point cloud is obtained through the random sampling of point clouds from the NURBS surface of the damaged blade. Also, due to the material missing areas on the surface of the simulated damaged blade, we expect to have fewer data points on the damaged blade point cloud with respect to the point cloud of the CAD model. Thus, the number of data points in the CAD point cloud and the synthetic point cloud is not equal. As depicted in Table 4, the computation times of CAD-to-scan non-rigid registration of the synthetic and denoised-simplified point clouds, respectively, are 22,812 and 888 s. As expected, the computation time for damage-free gDT construction of the simplified point cloud is significantly (more than 25 times) less than that of the synthetic point cloud. In each iteration of the non-rigid registration, the algorithm computes a unique correspondence and transformation matrix for each data point to project deformations to the CAD model [7]. Thus, by increasing the point cloud density, the computation time of the CAD-to-scan non-rigid registration algorithm increases. The results for the simulated point cloud data demonstrate that using the smoothed and down-sampled point cloud of the synthetic dataset effectively improves the accuracy and efficiency of generating the damage-free digital twin of the damaged blade.

### 4.2. Rigid and Non-Rigid Registration of Real Scanned Damaged Blade Point Clouds

**Figure 8:**
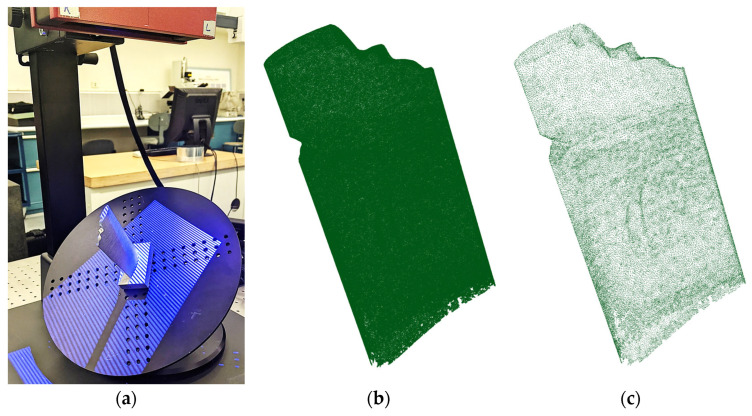
Experimental case study: (**a**) damaged blade being scanned by the structured-light 3D scanner, (**b**) corresponding raw point cloud containing measurement noises, outliers and a large number of data points (613,460 points), and (**c**) the denoised and simplified point cloud of the damaged blade obtained using the proposed method (47,160 points).

We also conducted an experimental case study to evaluate the performance of the rigid and non-rigid registration algorithms when applying the raw and simplified dataset of the real scanned point cloud of a damaged blade. Figure 8a,b show the damaged blade being scanned using an ATOS Core 200 (GOM, Braunschweig, Germany) structured-light 3D scanner and the scanned point cloud data, respectively. The raw scanned point cloud in Figure 8b contains 613,460 points. Figure 8c presents the denoised and simplified dataset derived from scan data using the proposed point cloud processing methodology. As can be seen in Figure 8c, the density of the simplified point cloud is significantly less than that of raw scan data. The simplified point cloud contains 47,160 points. The computation times required for denoising and downsampling of the raw scan data are, respectively, 232 and 74 s.

**Figure 9:**
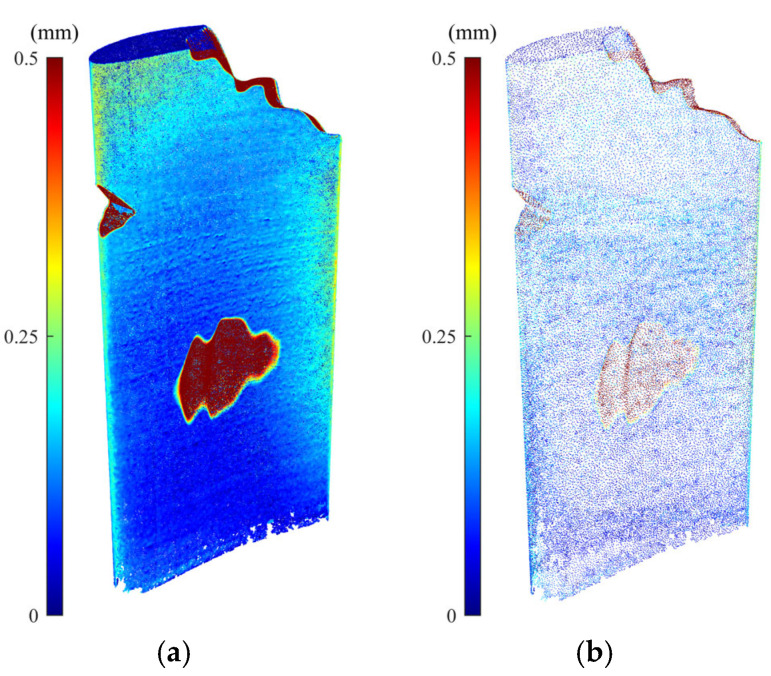
Error colormap of (**a**) scan data and (**b**) simplified point cloud with respect to the original CAD model after scan-to-CAD rigid registration.

**Figure 10:**
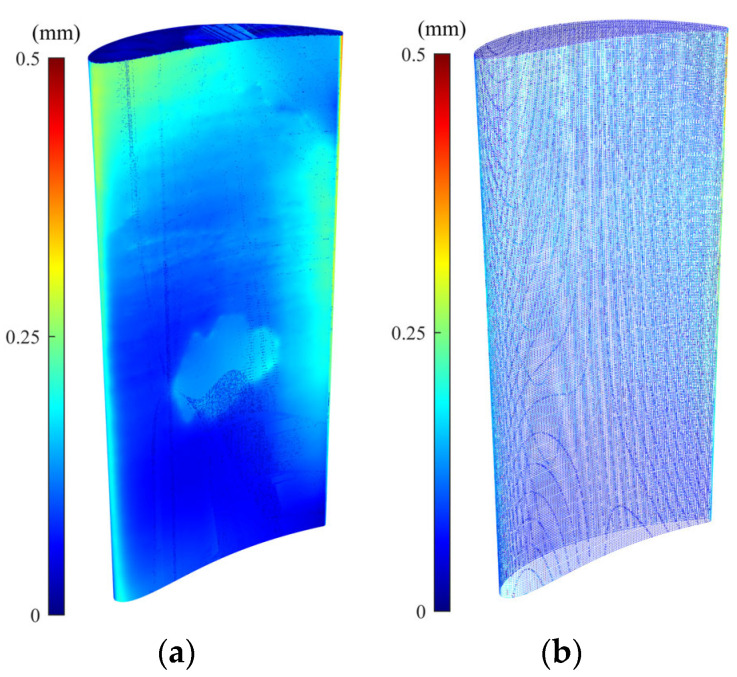
Error colormap of generated damage-free gDTs of (**a**) real scan data and (**b**) simplified point cloud with respect to the original CAD model after CAD-to-scan non-rigid registration.

Figure 9a,b, respectively, show the error colormap of raw scanned and simplified point clouds with respect to the CAD model after being aligned using the scan-to-CAD rigid registration algorithm. Figure 10, also, depicts the error colormap of the generated damage-free gDTs of raw scanned (Figure 10a) and simplified (Figure 10b) point clouds with respect to the nominal CAD model after CAD-to-scan non-rigid registration. The CAD models applied for damage-free gDT construction of raw scanned and simplified point clouds, respectively, contain 1,134,513 and 105,554 points. The error range of the colormap is capped at 0.5 mm for the sake of better visualization. Given the lack of a known reference for real scanned data, our evaluation of the performance of rigid and non-rigid registration algorithms focuses on error analysis within the undamaged regions of raw and simplified datasets of the damaged blade. Table 5 lists the average deviation of data points of undamaged regions of the raw scan data (Figure 9a) and simplified damaged blade (Figure 9b) from the CAD model and the damage-free gDTs. It is seen that, after rigid registration, the average deviation between the undamaged data points of the simplified point cloud and CAD model is 0.0196 mm less than those of raw scan data points. On the contrary, after non-rigid matching, the average deviation of the simplified point cloud from the damage-free gDT is 0.0018 mm more than the average deviation of raw scan data from its damage-free gDT. The results show that simplifying the point cloud of the damaged blade may lead to a potential alteration in fine details of scan data, which can affect the rigid and non-rigid registration results. It should be noted that due to a lack of a known reference model for real scan data, we cannot directly reach a conclusion on the effect of the proposed denoising and simplification method on the overall accuracy of rigid and non-rigid registrations. In our experimental case study, as can be seen in Table 5, employing the simplified point cloud leads to a noted decrease in the average deviation from the CAD model (after rigid registration) (0.0196 mm) and a slight increase in average deviation from damage-free gDT (after non-rigid matching) (−0.0018) with respect to the average deviation of raw scan data. Table 5 also presents the computation time for rigid and non-rigid registration of raw and simplified point clouds. As can be seen in Table 5, the computation time for rigid registration and non-rigid registration of the simplified point cloud is, respectively, 3.7 times and 43.2 times faster than the scan data. The results demonstrate that although using the proposed methodology we may lose a negligible detail of the underlying surface of point clouds, the computation time of registration and damage-free gDT construction of damaged blades can be dramatically reduced.

Although the current study validates the methodology on aero-engine blades, the underlying workflow is not restricted to this geometry. The denoising stage combines adaptive local surface fitting with measurement uncertainty-driven filtering, both of which adapt to local noise characteristics and local geometry rather than being tailored to blade-specific features. The subsequent Hausdorff distance-based simplification reduces point density while preserving critical details in high-curvature and boundary regions, again operating on generic geometric descriptors within the support domain of denoised points. This framework makes the approach potentially applicable to other free-form components with high geometric complexity, such as bladed disks (blisks), impellers, biomedical implants, and complex free-form molds. Nevertheless, empirical validation on such components remains an important step, and we plan to extend future work in this direction.

## 5. Conclusions

A new point cloud processing methodology for denoising and simplifying the raw scan data of damaged blades has been presented in this paper. The main contribution of the proposed method lies in employing a geometry-aware and automatic scheme for denoising, outlier removal, and simplification of the scanned point cloud within the uncertainty constraint of inspection data. In the denoising step, the algorithm benefits from an adaptive support domain to fit a weighted local least square plane/surface on the local neighborhood of each measured point under the measurement uncertainty constraint. The outliers are automatically detected and eliminated through analysis of projections of each scan data point within its expanded uncertainty interval. In the simplification step, the algorithm applies a Hausdorff distance-based region growing approach to search in the adaptive support domain of simplified data points to downsample the dense point cloud of the denoised dataset while preserving the underlying geometry of the surface as much as possible.

Implementation results on simulation and experimental case studies have demonstrated the effectiveness of the developed data processing approach for alignment and damage-free gDT construction of a damaged blade. Although the results show a negligible reduction in the accuracy of alignment of the simplified dataset because of losing some geometric details of the surface, the computation time of rigid and non-rigid alignment processes is considerably less than that of raw scan data with a huge number of data points.

The proposed point cloud simplification method facilitates the accurate and efficient inspection of in-service and damaged aero-engine blades using non-contact 3D scanning techniques. The presented approach also allows computing the repair volume for the remanufacturing of damaged blades with high efficiency and reasonable accuracy. Future research will focus on two directions:

- Integrating tolerance-aware, zone-specific simplification strategies that comply with aviation maintenance standards for aero-engine blades and evaluating the robustness of the methodology under different types of measurement noise and outliers.
- Validating the proposed workflow on additional complex free-form geometries to further demonstrate its generalizability beyond aero-engine blades.

## Figures and Tables

**Table 1:** Summary of neighborhood-based methods for smoothing and simplification of the raw scan data of blades.

**Method**	**Principle**	**Parameters/Features**	**Comments**
Bilateral Filtering [15,32,33]	Neighborhood-based smoothing method that combines spatial closeness and surface similarity (e.g., normals, intensity) to preserve edges while reducing noise	- Spatial kernel size - Range kernel size - Neighborhood size	- Preserves edges, reduces Gaussian-like noise, widely used. - Sensitive to parameter tuning and may over-smooth fine details.
Adaptive Bilateral Filtering [12,13,17]	Extends bilateral filter by adapting parameters per point (based on curvature, density, or local noise)	- Adaptive spatial kernel size - Adaptive range kernel size (noise/curvature-based) - Adaptive neighborhood size - Adaptation rule (curvature/density)	- Better preserves sharp features across varying densities, reduces over-smoothing. More robust than standard bilateral filtering. - Computationally expensive and requires pre-processing (curvature/density/noise estimation).
Clustering-based Simplification [13,29]	Groups nearby points into clusters (e.g., k-means and voxel clustering) and replaces each cluster with a representative point	- Number of clusters - Cluster classification threshold - Cluster representative rule (centroid/medoid/voxel center) - Cluster classification rule (distance, normal, curvature)	- Produces uniform reduction, simple to implement, good for large datasets. - Risk of oversimplification and parameter sensitivity.
Entropy-based Simplification [28,31]	Selects points that maximize local information content (curvature, saliency, entropy) to preserve geometric features	- Neighborhood size - Entropy threshold - Sampling ratio	- Effectively preserves sharp features and informative regions. - Computationally more expensive due to entropy evaluation.
Proposed method	Hybrid method combining surface fitting on adaptive local neighborhood for smoothing and a region growing-based approach for simplification	- Adaptive support domain - Outlier factor and weighting function - Weighted local least-squares fitting - Hausdorff distance-based region-growing	- Measurement uncertainty-driven - Threshold-free - Preserves geometry at high-curvature features - Computationally efficient - Requires pre-processing (local neighborhood/surface variation/normal).

**Table 2:** The average deviation of the actual, synthetic raw and simplified point cloud of the damaged blade from the CAD model in the design coordinate system and computation times of denoising and simplification steps.

Number of Points			Average Error from CAD (mm)			Computation Time (s)	
Actual	Synthetic	Simplified	Actual	Synthetic Raw	Synthetic Simplified	Denoising	Simplification
379,750	379,750	46,681	0.0391	0.0556	0.0402	44	53

**Table 3:** The average deviation of synthetic raw and simplified point clouds of the simulated damaged blade from the CAD model, and computation times for scan-to-CAD rigid registration process.

Average Deviation of Actual Damaged Blade from CAD Model (mm)	Average Error (mm)		Computation Time (s)	
	Synthetic Raw (Including Noise and Outliers)	Synthetic Simplified	Synthetic Raw (Including Noise and Outliers)	Synthetic Simplified
0.0391	0.0560	0.0412	769	72

**Table 4:** The average deviation of actual and constructed damage-free gDT models of the simulated damaged blade from the original CAD model, and computation times for the CAD-to-scan non-rigid registration process.

Average Deviation of Actual Damage-Free Digital Twin from CAD (Figure 5c) (mm)	Constructed Damage-Free gDT (mm)		Computation Time (s)	
	Synthetic Raw	Synthetic Simplified	Synthetic Raw	Denoised-Simplified
0.0145	0.0078	0.0115	22,812	888

**Table 5:** The average deviation of data points in undamaged regions of raw scanned and simplified point clouds of the real damaged blade, and computation times after scan-to-CAD rigid registration and CAD-to-Scan non-rigid registration process.

	Scan-to-CAD Rigid Registration		CAD-to-Scan Non-Rigid Registration	
	Deviation from CAD (mm)	Computation Time (s)	Deviation from Damage-Free gDT (mm)	Computation Time (s)
Raw scan data	0.1155	572	0.0122	40,258
Simplified dataset	0.0959	153	0.0141	932
Alteration	0.0196 (mm)	3.7 times faster	−0.0018 (mm)	43.2 times faster

## Data Availability

Data are contained within the article.

## Abbreviations

$Act(s_{j}^{\prime })$	Active data points in support domain of point $s_{j}^{\prime }$
c	Polynomial surface coefficients
CAI	Computer-Aided Inspection
$\bar{d}\left(p_{i}\right)$	Neighborhood distance of the point $p_{i}$
$\bar{D}\left(p_{i}\right)$	Neighborhood inner distance of the point $p_{i}$
$Inact(s_{j}^{\prime })$	Inactive data points in support domain of the point $s_{j}^{\prime }$
gDT	Geometric Digital Twin
MRO	Maintenance, Repair, and Overhaul
$M_{c}$	Covariance matrix
n + 1	Number of data points in scan point cloud
$OF\left(p_{i}\right)$	Outlier factor of the point $p_{i}$
NB($p_{i}$)	Local neighborhood of point $p_{i}$
${NB}_{P}=\{NB\left(p_{0}\right),NB\left(p_{1}\right),\ldots ,NB(p_{n})\}$	Local neighborhood set
$N_{P}=\{\vec{N_{p_{0}}},\vec{N_{p_{1}}},\ldots ,\vec{N_{p_{n}}}\}$	Normal vector set
P={$p_{0},p_{1},\ldots ,p_{n}$}	Raw scan data
$P_{D}=${$s_{0},s_{1},\ldots ,s_{m}$}	Denoised dataset
$P_{s}=${$s_{0}^{\prime },s_{1}^{\prime },\ldots ,s_{t}^{\prime }$}	Simplified dataset
PCA	Principal Component Analysis
RMSE	Root Mean Square error
${Res}_{P_{D}}$	Reserve dataset
$R_{p_{i}}=\{p_{i}^{0},p_{i}^{1},p_{i}^{2},\ldots ,p_{i}^{j},\ldots ,p_{i}^{r-1}\}$	Projected coordinates of the point $p_{i}$
${SUP}_{D}=\{{SupD(s}_{0}),\ldots ,{SupD(s}_{m})\}$	Support domain of denoised dataset
${sv(p}_{i})$	Surface variation around the point $p_{i}$
${SV}_{P}=\{sv\left(p_{0}\right),sv\left(p_{1}\right),\ldots ,sv\left(p_{n}\right)\}$	Surface variation set
${SupD(p}_{i})$	Support domain of the point $p_{i}$
TC	Territory Claiming algorithm
u	Standard uncertainty of data
WLLS	Weighted local least-squares
$w\left(p_{i}\right)$	Weight of the point $p_{i}$
$\hat{f}\left(x,y\right)$	Polynomial surface
$\sigma_{out}$	Standard deviation of outlier factor values
δ	Surface variation threshold
$\lambda_{l}\left(l=0,1,2\right)$	Eigenvalues of covariance matrix
